# Histone deacetylase HDAC2 silencing prevents endometriosis by activating the HNF4A/ARID1A axis

**DOI:** 10.1111/jcmm.16835

**Published:** 2021-09-29

**Authors:** Hong Mai, Yan Liao, Sufang Luo, Kaiyi Wei, Feng Yang, Haijuan Shi

**Affiliations:** ^1^ Department of Gynecology The Second Affiliated Hospital of Guangxi Medical University Nanning China

**Keywords:** apoptosis, ARID1A, deacetylation, endometriosis, HDAC2, histone deacetylase, HNF4A, proliferation

## Abstract

Endometriosis is the most major cause of chronic pelvic pain in women of reproductive age. Moreover, the involvement of histone deacetylase 2 (HDAC2) has been identified in endometriosis. However, the specific mechanism of HDAC2 remains to be further elusive. Therefore, this study was designed to explore the mechanism of HDAC2 orchestrating hepatocyte nuclear factor 4α/AT‐rich interactive domain 1A (HNF4A/ARID1A) axis in endometriosis. Endometriosis cell line hEM15A and clinical endometriosis tissues were obtained, followed by gain‐ and loss‐of‐function assays in hEM15A cells. HDAC2, HNF4A and ARID1A expression was detected by immunohistochemistry and Western blot analysis. Cell viability was determined by Cell Counting Kit‐8 Assay, invasion by Transwell assay and apoptosis by flow cytometry. HDAC2 enrichment in HNF4A promoter region and HNF4A enrichment in ARID1A promoter region was detected through chromatin immunoprecipitation. Mouse models of endometriosis were established, followed by immunohistochemistry of Ki‐67 expression and TUNEL staining of apoptosis in ectopic tissues. HDAC2 was upregulated but HNF4A and ARID1A were downregulated in endometriosis tissues. HDAC2 inhibited HNF4A expression by deacetylation, and HNF4A was enriched in ARID1A promoter region to activate ARID1A. Silencing HDAC2 or overexpressing HNF4A or ARID1A diminished the viability and invasion and augmented the apoptosis of hEM15A cells. HDAC2 silencing reduced the area and weight of endometriosis tissues, suppressed endometriosis cell proliferation and accelerated endometriosis cell apoptosis. The inhibitory action of silencing HDAC2 via HNF4A/ARID1A axis was reproduced in mouse models. Collectively, HDAC2 silencing might upregulate HNF4A via repression of deacetylation to activate ARID1A, thus preventing the occurrence of endometriosis.

## 1 INTRODUCTION

Endometriosis is defined as the presence of endometrial glands and stroma outside the normal location of the endometrium.^1^ As an oestrogen‐dependent gynaecological disease, endometriosis possesses the feature of the existence and growth of ectopic endometrial tissues and usually correlates to inflammation, severe and chronic pain, and infertility.^2^ Additionally, endometriosis is the most principled cause of chronic pelvic pain in women of reproductive age and is closely associated with sustained episodes of ovulation, menstruation and circulating steroid hormones.^3^ Unfortunately, current treatments, depending on the primary indication (infertility or pelvic pain), are limited to surgical and hormonal treatments and analgesics with many adverse effects that rarely provide long‐term relief.^4^ Hence, it is necessary to explore the molecular mechanism underlying endometriosis for better and safer therapies.

Histone deacetylase 2 (HDAC2) is one of the class I histone deacetylase, which manipulates epigenetic landscape through histone modification.^5^ It has been reported that HDAC2 is capable of orchestrating human smooth muscle cells of the uterus.^6^ Furthermore, a prior study has elucidated the upregulation of HDAC2 in endometriosis.^7^ According to a literature, HDAC2 overexpression contributes to the deacetylation of the hepatocyte nuclear factor 4α (HNF4A) transcription factor during Alzheimer's disease.^8^ Importantly, the involvement of HNF4A has been noted in the pathogenesis of endometriosis.^9^ It has been documented that HNF4A can act as a transcription factor to promote the expression of downstream genes.^10^ However, the relationship between HNF4A and AT‐rich interactive domain 1A (ARID1A) has been rarely investigated, which warranted our research to study their relationship. AT‐rich interaction domain 1A (ARID1A) is a 250 kD switch/sucrose non‐fermentable chromatin remodelling complex subunit with known tumour suppressor function and is associated with both endometriosis and orchestration of endometrial receptivity.^11^ Intriguingly, ARID1A downregulation has been elaborated to be involved in the pathogenesis of endometriosis.^12^ In this context, we speculated that the network of HDAC2, HNF4A and ARID1A might be correlated with the pathogenesis of endometriosis. Therefore, tissues, cells and animal experiments were implemented in this research to verify this speculation, thus providing a novel candidate target for endometriosis treatment.

## 2 MATERIALS AND METHODS

### 2.1 Ethics statement

This study was performed with approval of the Ethics Committee of The Second Affiliated Hospital of Guangxi Medical University by conforming to the *Declaration of Helsinki*. All participants or their guardians provided signed informed consent prior to enrolment. The animal experiments were approved by the animal ethics committee of The Second Affiliated Hospital of Guangxi Medical University and conformed to the animal ethics standards.

### 2.2 Study subjects

A total of 40 patients with ovarian endometriosis were selected in The Second Affiliated Hospital of Guangxi Medical University from January 2017 to December 2019. All patients were diagnosed as endometriosis by pathology. Among patients, their age ranged from 27 to 54 years, with a mean age of 38.88 ± 7.26 years. In addition, 30 patients who underwent hysterectomy due to hysteromyoma were enrolled as the positive control group, with a mean age of 39.80 ± 6.96 years. All patients were not treated with sex hormone nor anti‐endometriosis drugs in the first three months.

### 2.3 Immunohistochemical staining

The tissue specimens were embedded in paraffin, sectioned, dewaxed and hydrated. The sections were washed in 3% methanol H_2_O_2_ for 20 min, followed by antigen retrieval with citrate buffer in pressure cooker (2 min at 100℃ and 5 min at room temperature). Normal goat serum blocking solution (C‐0005, Shanghai Haoran Biotechnology Co., Ltd.) was dripped to the tissue sections. Then, the sections were placed at room temperature for 20 min. The liquid on the sections was dried. Primary rabbit anti‐human antibodies to HDAC2 (12922‐3‐AP, 1:200, Proteintech), HNF4A (ab92378, 1:500, Abcam), Ki‐67 (ab16667, 1:200, Abcam), and ARID1A (ab182560, 1:1000, Abcam) were added to the sections for overnight culture at 4℃, and the secondary antibody (ab6785, 1:1000, Abcam) was added. After incubation, protein working solution (0343‐10000U, Imun Biotechnology Co., Ltd.) was added into the sections and placed at 37℃ for 20 min. The sections were developed with diaminobenzidine (DAB; ST033, Whiga Biosmart Co., Ltd.), counterstained with haematoxylin (PT001, Shanghai Bogoo Biological Technology Co., Ltd.) for 1 min and blued with 1% ammonia water. The sections were observed and photographed under a microscope after sealing. Five visual fields were randomly selected from each section to observe and analyse the statistics.

### 2.4 Bioinformatics analysis

Endometriosis‐related gene expression dataset GSE37837 was obtained through Gene Expression Omnibus database (https://www.ncbi.nlm.nih.gov/gds), including 18 normal samples and 18 endometriosis samples. The genes with |logFoldChange| > 1.5 and *p* < 0.05 were selected as significantly upregulated genes through differential analysis of GSE37837 through R language ‘limma’ package (https://bioconductor.org/packages/limma/). In GeneCards (https://www.genecards.org/), ‘endometriosis’ was retrieved to select the top 1000 genes, which were intersected with the significantly upregulated genes. The correlation between the intersected gene and endometriosis was analysed by phenolyzer (http://phenolyzer.wglab.org/). Based on the existing literature and the results of phenolyzer, the key genes were identified, and the downstream regulatory pathways were predicted. Gene Expression Profiling Interactive Analysis (GEPIA; http://gepia2.cancer‐pku.cn/#index) was used to analyse the combined data of Uterine Corpus Endometrial Carcinoma (UCEC) and Genotype‐Tissue Expression in The Cancer Genome Atlas database (https://portal.gdc.cancer.gov/) to obtain HDAC2 expression. Pearson's correlation was calculated based on the expression data of endometriosis samples in GSE37837 to determine the correlation between HDAC2 and HNF4A expression, and between HNF4A and ARID1A expression. Totally, 1500 significant co‐expression relationships of HNF4A were predicted, and the co‐expression relationship between HNF4A and ARID1A was verified by Multi Experiment Matrix (MEM; https://biit.cs.ut.ee/mem/index.cgi). The promoter sequence of ARID1A was obtained from University of California Santa Cruz (UCSC; http://genome.ucsc.edu/). hTFtarget (http://bioinfo.life.hust.edu.cn/hTFtarget#!/) was utilized to predict the binding site of HNF4A to ARID1A promoter.

### 2.5 Cell screening, culture and transfection

Endometriosis cell line hEM15A was purchased from Cobioer Biotechnology Co., Ltd. hEM15A cells were cultured in an incubator (Thermo Fisher Scientific Inc.) under the condition of 37℃ and 5% CO_2_ with Roswell Park Memorial Institute 1640 medium (Gibco) containing 10% foetal bovine serum (FBS, Gibco), 10 μg/ml streptomycin and 100 U/ml penicillin. The cells were trypsinized when they were in logarithmic phase. The cells were seeded into 6‐well plates at 1 × 10^5^ cells per well and cultured for 24 h. When cell confluence was about 60%, the cells were transfected according to the instructions of Lipofectamine 2000 (Invitrogen Inc.). The groups were as follows: short hairpin RNA (sh)‐negative control (NC) (transfection of 5′‐GGGUGAACUCACGUCAGAA‐3′ sequence), sh‐HDAC2‐1 (transfection of 5′‐UCAACCUAGUGCUGUGGUAUU‐3′ sequence), sh‐HDAC2‐2 (transfection of 5′‐CCAATGAGTTGCCATATA‐3′ sequence), sh‐HDAC2‐3 (transfection of 5′‐CAATGAGTTGCCATA TAAT‐3′ sequence), overexpression (oe)‐NC and oe‐ARID1A. The expression plasmids were purchased from Shanghai GenePharma Co. Ltd. at a concentration of 50 ng/ml.

### 2.6 Reverse transcription‐quantitative polymerase chain reaction (RT‐qPCR)

TRIzol (Invitrogen) was applied to extract total RNA from tissues and cells. A NanoDrop2000 micro ultraviolet spectrophotometer (1011U, NanoDrop Technologies) was adopted to detect the concentration and purity of total RNA. As per the manuals of a PrimeScript RT reagent Kit (RR047A, Takara), cDNA was generated from RNA. Primers for HNF4A and ARID1 were designed and synthesized by Takara Company (Table S1). Real‐time fluorescent qPCR was implemented in ABI7500 qPCR instrument (7500, ABI). The relative transcription level of target gene was calculated by 2^−ΔΔCT^ method with glyceraldehyde‐3‐phosphate dehydrogenase (GAPDH) as a normalizer.

### 2.7 Western blot analysis

The cultured cells were collected and lysed with enhanced Radio‐Immunoprecipitation assay cell lysis buffer containing protease inhibitor (Boster Biological Technology Co., Ltd.). Then, the bicinchoninic acid protein quantitative kit (Boster Biological Technology Co., Ltd.) was adopted to estimate the protein concentration. The protein was transferred to a polyvinylidene fluoride membrane after 10% sodium dodecyl sulphate polyacrylamide gel electrophoresis. After 2‐h sealing with 5% bovine serum albumin at room temperature, the membrane was probed with primary rabbit anti‐human antibodies (Abcam) to HDAC2 (ab32117, 1:2000), HNF4A (ab92378, 1:1000), ARID1A (ab182560, 1:1000), and GAPDH (ab8245, 1:1000) overnight at 4℃. Horseradish peroxidase‐labelled goat anti‐rabbit immunoglobulin G (IgG) (ab6721, 1:5000, Abcam) was added into the membrane. After 1‐h incubation at room temperature, the membrane was developed with electrogenerated chemiluminescence working solution (EMD Millipore) and exposed using a Bio‐Rad automatic imager. ImageJ analysis software was adopted to quantify the grey value of each band in the Western blot image with GAPDH as a normalizer.

### 2.8 Cell counting kit (CCK)‐8 experiment

A CCK‐8 assay (CK04, Dojindo Laboratories) was employed to detect cell viability. Cells were seeded into a 96‐well plate at 3 × 10^3^ cells/well. After treatment, 10 μl CCK‐8 solution and 100 μl fresh medium were added into each well at 0, 24, 48 and 72 h, and incubated at 37℃ for 2 h. The optical density value was detected at 450 nm using a microplate reader (Bio‐Rad 680, Bio‐Rad Laboratories). Each sample had five duplicated wells.

### 2.9 Transwell invasion experiment

Before the experiment, the filter membrane was coated with 50 μl Matrigel (Sigma‐Aldrich Chemical Company). The cells in logarithmic growth phase were starved for 24 h and detached on the next day to make the final concentration of cells 2 × 10^5^ cells/ml. The 0.2 ml suspension was added into Transwell upper chamber. In the lower chamber, 700 μl Dulbecco's Modified Eagle Medium containing 10% FBS was added. The chamber was cultured in a cell incubator containing 5% CO_2_ at 37℃. After 24 h, the Transwell chamber was harvested, fixed with methanol and stained with 0.1% crystal violet. The number of stained cells was counted under an inverted microscope (XDS‐800D, Shanghai Caikon Optical Instrument Co., Ltd.). Five visual fields were randomly selected for counting, and the number of cells was expressed as mean.

### 2.10 Flow cytometry

After 48 h of transfection, hEM15A cells were centrifuged at 500–1000 *g* for 5 min, and the medium was discarded. hEM15A cells were washed once with 3 mL phosphate buffer saline (PBS). After PBS was removed by centrifugation, cells were fixed at 4℃ with 70% ethanol precooled by ice for 1–2 h. Centrifugation was implemented to discard the fixative, and cells were resuspended with 3 ml PBS for 5 min. The suspension was filtered once with a 400‐mesh sieve and centrifuged at 500–1000 *g* for 5 min, followed by discarding of PBS. The apoptotic cells were double‐stained with propidium iodide and Annexin V‐Fluorescein Isothiocyanate Apoptosis Detection Kit I (BD Biosciences). The apoptosis was detected by a flow cytometer (Bio‐Rad ZE5, Bio‐Rad Laboratories).

### 2.11 Chromatin immunoprecipitation (ChIP) assay

A ChIP kit (EMD Millipore) was conducted to study the protein enrichment of HNF4A and ARID1A promoters. The cells in the logarithmic growth phase were added with 1% formaldehyde and fixed at room temperature for 10 min to cross‐link DNA and protein. After crosslinking, the samples were randomly broken by ultrasonic treatment for 15 cycles at the interval of 10 s with 10 s for each time. After centrifugation at 4℃ and 34017*g* (some DNA fragments as input), the supernatant was collected and aliquoted into two tubes which were respectively supplemented with IgG (ab18413, 1:50) of normal mice as NC antibody and the specific antibody of target protein (all antibodies from Abcam). After overnight incubation at 4℃, the endogenous DNA protein complex was precipitated by protein agarose/Sepharose. After centrifugation, the supernatant was removed and the non‐specific complex was washed and decrosslinked at 65℃ overnight. Then, DNA fragments were extracted and purified with phenol/chloroform. The aggregation level of HNF4A and ARID1A was detected by RT‐qPCR by using IgG as internal reference.

### 2.12 Establishment of mouse model of endometriosis

A total of 24 NOD/SCID mice aged 5–6 weeks (8 mice were used for uterus donation modelling) were purchased from Model Animal Research Center of Nanjing University and injected subcutaneously with a micro‐injector. The mice were assigned into two groups including sh‐NC group and sh‐HADC2 group (8 mice in each group). Both the donor and recipient mice were injected with 100 mg/kg oestrogen (Santa Cruz Biotechnology Inc.) once a week 14 days before the establishment of the model. One donor mouse uterus was used by two recipient mice. After the mice were euthanized, the uterus was found, and the mesometrium was separated and put into the sterile plate containing normal saline. The tissues were cut into 1 × 1 mm pieces, the tissue fragments were injected into the peritoneal cavity of the recipient mice (100 μl/mouse), and the culture was continued. After the model establishment was started, sh‐NC vector and sh‐HADC2 vector were injected intraperitoneally into mice from day 0 once every three days (100 μl/mice) for four weeks. Four weeks later, the mice were euthanized and mice were dissected to attain the ectopic uterine tissues. The weight and area of ectopic uterine tissues were measured and photographed.

### 2.13 Terminal deoxyribonucleotidyl transferase (TdT)‐mediated 2′‐deoxyuridine 5′‐triphosphate‐biotin nick end‐labelling (TUNEL) staining

After dewaxing, the paraffin‐embedded sections were immersed in 0.85% NaCl for 5 min, in PBS for 5 min and in 4% paraformaldehyde for 15 min in turn. 100 μl of 20 μg/ml proteinase K was added to completely cover the tissues for 20‐min incubation at room temperature. The sections were immersed in PBS for 5 min, in 4% paraformaldehyde for 5 min and in PBS for 5 min, which was repeated once. The excess liquid was removed, and 100 μl equilibrium solution was added to sections for 5‐min incubation at room temperature. The equilibrium solution was removed, and the rTdT reaction solution was added. The samples were transferred into a wet box and incubated at 37℃ for 1 h. After the plastic cover glass was removed, the samples were stained for 15 min in a dyeing tank containing 2 × sodium chloride‐sodium citrate. After completion, the samples were put into 0.3% hydrogen peroxide water prepared by PBS for 2 min, coloured with DAB, and sealed with neutral resin.

### 2.14 Statistical analysis

SPSS 21.0 (IBM Corp.) was adopted to analyse the data of this study. The measurement data were expressed as mean ± standard deviation. Unpaired *t* test was applied for comparison between the two groups, one‐way analysis of variance (anova) for comparison among multiple groups and two‐way anova for comparison between groups at different time, followed by Tukey's post hoc test. *p* < 0.05 meant that the difference was statistically significant.

## 3 RESULTS

### 3.1 HDAC2 was highly expressed in endometriosis

**FIGURE 1:**
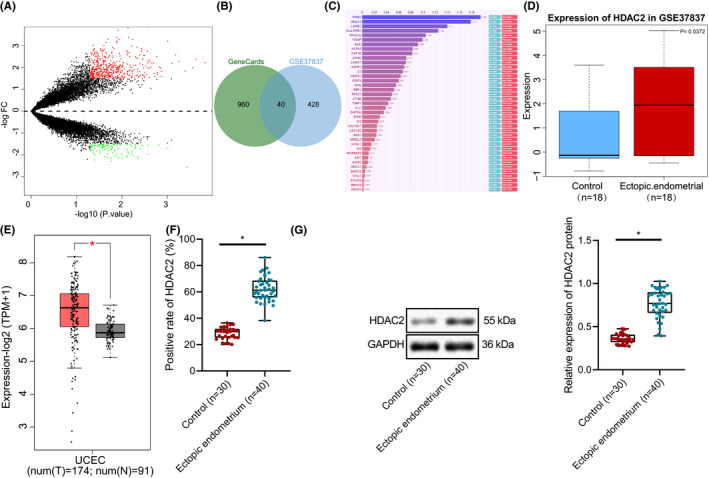
HDAC2 expression is high in endometriosis. (A) The volcano map of gene expression in GSE37837. The black dot indicates the genes with no significant difference, the red dot indicates the significantly upregulated gene, and the green dot indicates the significantly downregulated gene. (B) Venn map of 40 intersected genes between the significantly upregulated genes of GSE37837 and the gene retrieved by GeneCards. (C) The importance of genes in endometriosis analysed by phenolyzer. When the length of the column was longer, the importance was higher. (D) HDAC2 expression in GSE37837. The left blue box showed the expression in normal samples, and the right red box showed the expression in endometriosis samples. The data were expressed as median ± interquartile range. (E) GEPIA of the expression of HDAC2 in UCEC. The left red box was the expression in UCEC, and the right grey box was the expression in the control group. The data were expressed as median ± interquartile range. *p < 0.05. (F) The expression of HDAC2 protein in normal tissues (*n* = 30) and endometriosis tissues (*n* = 40) detected by immunohistochemistry. The data were expressed as median ± interquartile range. (G) Western blot analysis of the expression of HDAC2 protein in normal tissues (*n* = 30) and endometriosis tissues (*n* = 40). The data were expressed as median ± interquartile range. *p < 0.05 vs. the control group. The experiment was repeated three times independently

In order to deeply understand the molecular mechanism of endometriosis, bioinformatics analysis was conducted to screen the differentially expressed genes in endometriosis. A total of 468 upregulated genes in endometriosis were obtained by differential analysis of GSE37837 (Figure 1A). The top 1000 genes related to ‘endometriosis’ were obtained from GeneCards and then were intersected with the upregulated genes in GSE37837, which generated 40 important genes of endometriosis (Figure 1B). The results of phenolyzer analysis showed that ITGB1, HDAC2 and LAMB1 were the top three important genes in endometriosis (Figure 1C).

In order to explore the effect of HDAC2 on endometriosis, HDAC2 expression data were extracted from GSE37837 to draw the box plot, which revealed HDAC2 high expression in endometriosis (Figure 1D). GEPIA also displayed that HDAC2 was highly expressed in UCEC (Figure 1E). Western blot analysis and immunohistochemistry described that the protein expression of HDAC2 in endometriosis tissues was significantly higher than that in normal tissues (Figure 1F–G). These results indicated that HDAC2 was upregulated in endometriosis.

### 3.2 Silencing HDAC2 inhibited proliferation and migration and promoted apoptosis of endometriosis cells

**FIGURE 2:**
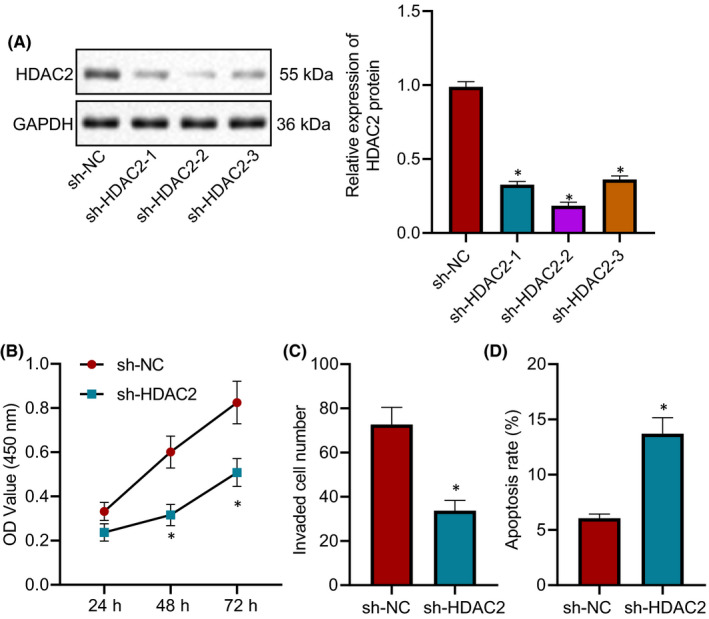
Silencing HDAC2 decreases the proliferation and invasion and increases the apoptosis of endometriosis cells. (A) The expression of HDAC2 in hEM15A cells of each group detected by Western blot analysis. (B) The proliferation of hEM15A cells in each group detected by CCK‐8 method. (C) The invasion of hEM15A cells in each group measured by Transwell assay. (D) The apoptosis of hEM15A cells in each group detected by flow cytometry. Data were shown as median ± standard error of mean (*n* = 3). *p < 0.05 vs. the sh‐NC group. The experiment was repeated three times independently

In order to explore the effect of HDAC2 on endometriosis cells, HDAC2 was silenced in endometriosis cell line hEM15A. Through Western blot analysis, it was found that compared with the sh‐NC group, the expression of HDAC2 protein in the sh‐HDAC2‐1, sh‐HDAC2‐2 and sh‐HDAC2‐3 groups was significantly decreased (Figure 2A). Based on the results of CCK‐8 (Figure 2B), Transwell assay (Figure 2C), and flow cytometry (Figure 2D), hEM15A cell viability and invasion were suppressed and hEM15A cell apoptosis was promoted in the sh‐HDAC2 group than those in the sh‐NC group. Taken together, silencing HDAC2 repressed the proliferation and invasion and induced the apoptosis of endometriosis cells.

### 3.3 HDAC2 inhibited HNF4A expression by deacetylation

**FIGURE 3:**
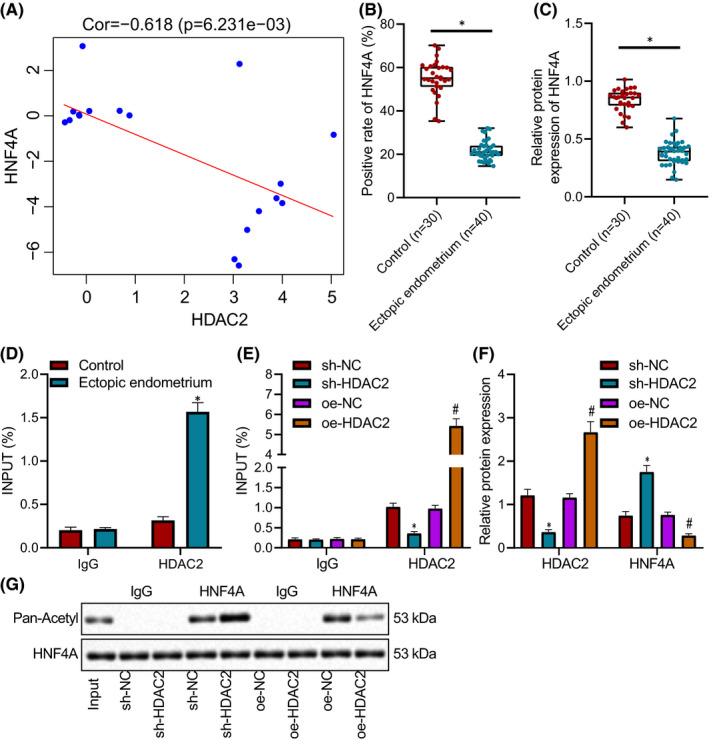
HDAC2 reduces HNF4A expression through deacetylation. (A) Pearson correlation map of HDAC2 and HNF4A expression in GSE37837. (B) The expression of HNF4A protein in normal tissues (*n* = 30) and endometriosis tissues (*n* = 40) determined by immunohistochemistry. The data were expressed as median ± interquartile range. (C) The expression of HNF4A protein in normal tissues (*n* = 30) and endometriosis tissues (*n* = 40) measured by Western blot analysis. The data were expressed as median ± interquartile range. (D) The enrichment of HDAC2 in the promoter region of HNF4A in normal tissues (*n* = 30) and endometriosis tissues (*n* = 40) measured by ChIP. (E) The enrichment of HDAC2 in the promoter region of HNF4A of each group detected by ChIP. (F) The expression of HDAC2 and HNF4A in each group assessed by Western blot analysis. (G) HNF4A acetylation level following IP. Data were shown as median ± standard error of mean (*n* = 6). * *p* < 0.05 vs. the sh‐NC group; ^#^ *p* < 0.05 vs. the oe‐NC group. The experiment was repeated three times independently

It has been shown that HDAC2 can affect the expression of HNF4A through deacetylation.^8^ In order to explore the effect of HDAC2 on the expression of HNF4A during endometriosis, we first found a significant negative correlation between HDAC2 and HNF4A expression in endometriosis by GSE37837 (Figure 3A). Moreover, Western blot analysis and immunohistochemistry depicted that the expression of HNF4A protein in endometriosis tissues was significantly lower than that in normal tissues (Figure 3B,C). Further ChIP analysis documented that HDAC2 increased significantly in the promoter region of HNF4A in endometriosis tissues compared with normal tissues (Figure 3D). Compared with the sh‐NC group, HDAC2 enrichment in HNF4A promoter region was significantly decreased in the sh‐HDAC2 group, and compared with the oe‐NC group, HDAC2 enrichment in HNF4A promoter region was substantially increased in the oe‐HDAC2 group (Figure 3E). It was detected by Western blot analysis that compared with the sh‐NC group, HDAC2 expression in the sh‐HDAC2 group was conspicuously diminished, whereas HNF4A expression was augmented, which were opposite in the oe‐HDAC2 group in contrast to the oe‐NC group (Figure 3F). Following HNF4A IP assay, acetylation level of HNF4A was detected by Western blot analysis, results of which revealed significantly enhanced acetylation level of HNF4A in presence of HDAC2 yet suppressed acetylation level of HNF4A when HDAC2 was overexpressed (Figure 3G). In summary, HDAC2 caused HNF4A downregulation through deacetylation.

### 3.4 Silencing HDAC2 depressed the proliferation and invasion and accelerated the apoptosis of endometriosis cells by activating HNF4A

**FIGURE 4:**
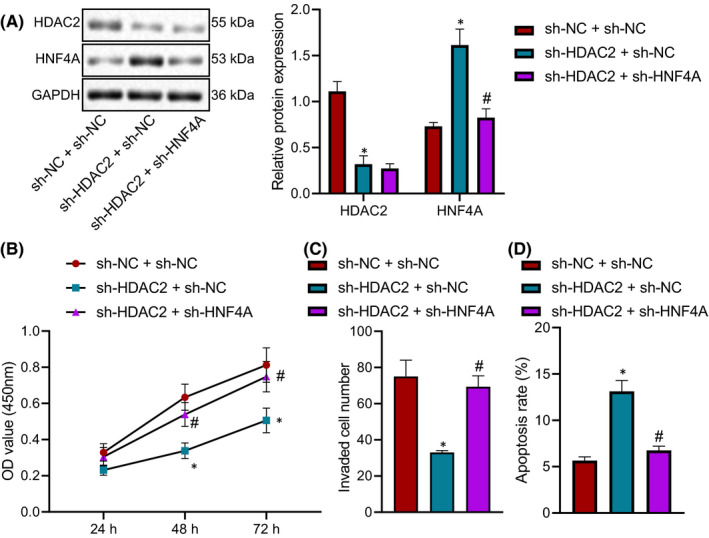
HDAC2 silencing elevates HNF4A expression to slow the proliferation and invasion and facilitate the apoptosis of endometriosis cells. (A) The expression of HDAC2 and HNF4A in hEM15A cells of each group evaluated by Western blot analysis. (B) The viability of hEM15A cells determined by CCK‐8 method. (C) hEM15A cell invasion in each group measured by Transwell assay. (D) Apoptosis of hEM15A cells in each group detected by flow cytometry. Data were shown as median ± standard error of mean (*n* = 3). *p < 0.05 vs. the sh‐NC + sh‐NC group; ^#^ *p* < 0.05 vs. the sh‐HDAC2 + sh‐NC group. The experiment was repeated three times independently

In order to explore the effect of the HDAC2/HNF4A axis on endometriosis cells, HDAC2 and HNF4A were silenced in the endometriosis cell line hEM15A alone or in combination. Western blot analysis demonstrated that compared with the sh‐NC + sh‐NC group, HDAC2 protein expression in the sh‐HDAC2 + sh‐NC group was potently reduced, and HNF4A protein expression was evidently enhanced. Compared with the sh‐HDAC2 + sh‐NC group, HNF4A protein expression in the sh‐HDAC2 + sh‐HNF4A group was appreciably declined (Figure 4A). CCK‐8 (Figure 4B), Transwell assay (Figure 4C), and flow cytometry (Figure 4D) depicted that compared with the sh‐NC + sh‐NC group, hEM15A cell viability and invasion in the sh‐HDAC2 + sh‐NC group were appreciably inhibited, and hEM15A cell apoptosis was facilitated, which were opposite in the sh‐HDAC2 + sh‐HNF4A group when compared to the sh‐HDAC2 + sh‐NC group. Collectively, silencing HDAC2 could repress the proliferation and invasion and augmented the apoptosis of endometriosis cells by upregulating HNF4A.

### 3.5 Proliferation and invasion of endometriosis cells were inhibited, and cell apoptosis was promoted by activating the HNF4A/ARID1A axis

**FIGURE 5:**
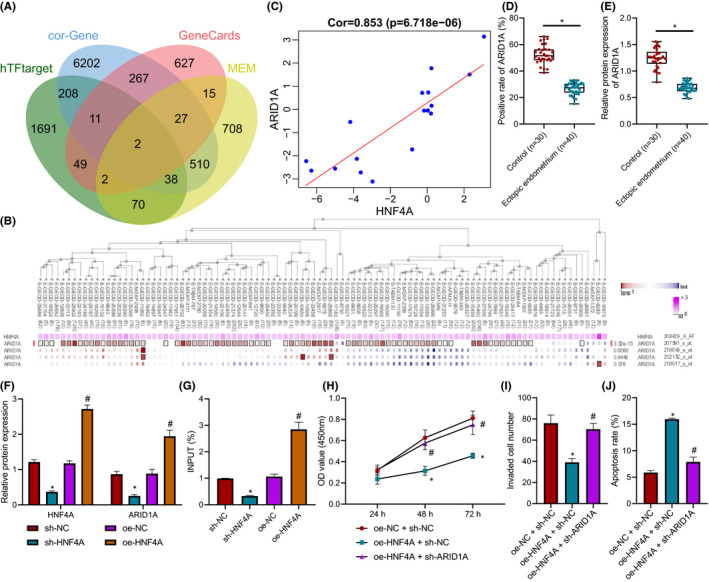
HNF4A upregulation increases ARID1A expression to suppress the proliferation and invasion and accelerates the apoptosis of endometriosis cells. (A) Venn map of HNF4A target genes predicted by hTFtarget, and HNF4A‐related genes in GSE37837, endometriosis‐related genes predicted by GeneCards, and genes co‐expressed with HNF4A predicted by MEM. The intersected genes were ARID1A and F2. (B) MEM predicting the co‐expression graph of HNF4A and ARID1A. The horizontal axis of ARID1A was the red dot, which indicated that there was a positive correlation in this data set, and otherwise it was a negative correlation. (C) Pearson correlation of HNF4A and ARID1A expression in GSE37837. (D) The expression of ARID1A protein in normal tissues (*n* = 30) and endometriosis tissues (*n* = 40) assessed by immunohistochemistry. The data were expressed as median ± interquartile range. (E) The expression of ARID1A protein in normal tissues (*n* = 30) and endometriosis tissues (*n* = 40) detected by Western blot analysis. The data were expressed as median ±interquartile range. (F) The expression of HNF4A and ARID1A in hEM15A cells of each group evaluated by Western blot analysis. (G) The enrichment of HNF4A in the promoter region of ARID1A in each group measured by ChIP. (H) The proliferation of hEM15A cells detected by CCK‐8 method. (I) Transwell assay to determine the invasion of hEM15A cells. (J) The apoptosis of hEM15A cells assessed by flow cytometry. Data were shown as median ± standard error of mean (*n* = 3). *p < 0.05 vs. the oe‐NC + sh‐NC group; ^#^ *p* < 0.05 vs. the oe‐HNF4A + sh‐NC group. The experiment was repeated three times independently

Then, we explored the downstream mechanism of HNF4A in endometriosis. Firstly, 2071 downstream genes of HNF4A were predicted by hTFtarget. Through the co‐expression analysis of MEM, the top 1500 significant co‐expressions were screened out, and 1373 co‐expression genes were obtained. In order to confirm that the downstream gene was endometriosis‐related gene, the Pearson correlation between HNF4A and other genes in endometriosis samples of GSE37837 was analysed, which screened out 7265 genes with |cor| > 0.8 and *p* < 0.05. Meanwhile, 1000 endometriosis‐related genes were predicted through GeneCards. Then, these predicted results were intersected, which found that the most critical genes were ARID1A and F2 (Figure 5A).

A previous study has reported that ARID1A is poorly expressed in endometriosis.^12^ The promoter sequence of ARID1A was obtained through UCSC to verify the relationship between HNF4A and ARID1A. Several binding sites of HNF4A to ARID1A promoter were predicted by hTFtarget (Table S2). The co‐expression graph of HNF4A and ARID1A was obtained from MEM (Figure 5B). The co‐expression diagram exhibited that HNF4A was positively correlated with ARID1A. Correlation map drawn based on analysis in microarray dataset GSE37837 revealed a positive correlation between HNF4A and ARID1A expression in endometriosis tissues (Figure 5C).

Next, Western blot analysis and immunohistochemistry showed that the protein expression of ARID1A in endometriosis tissues was strikingly lower than that in normal tissues (Figure 5D,E). After HNF4A was silenced and overexpressed in the endometriosis cell line hEM15A, Western blot analysis displayed that in comparison with the sh‐NC group, the expression of HNF4A and ARID1A in the sh‐HNF4A group was remarkably decreased, whereas the protein expression of HNF4A and ARID1A in the oe‐HNF4A group was prominently increased compared with the oe‐NC group (Figure 5F). Based on ChIP results, in contrast to the sh‐NC group, HNF4A enrichment in ARID1A promoter region was severely reduced in the sh‐HNF4A group but was markedly elevated in the oe‐HNF4A group compared with oe‐NC group (Figure 5G).

According to CCK‐8 (Figure 5H), Transwell assay (Figure 5I) and flow cytometry (Figure 5J), hEM15A cell viability and invasion were noticeably inhibited and hEM15A cell apoptosis was observably enhanced in the oe‐HNF4A + sh‐NC group versus the oe‐NC + sh‐NC group, which was reversed in the oe‐HNF4A + sh‐ARID1A group in comparison to the oe‐HNF4A + sh‐NC group. Conclusively, the activation of the HNF4A/ARID1A axis inhibited the proliferation and invasion and promoted the apoptosis of endometriosis cells.

### 3.6 Silencing HDAC2 inhibited endometriosis in mice

**FIGURE 6:**
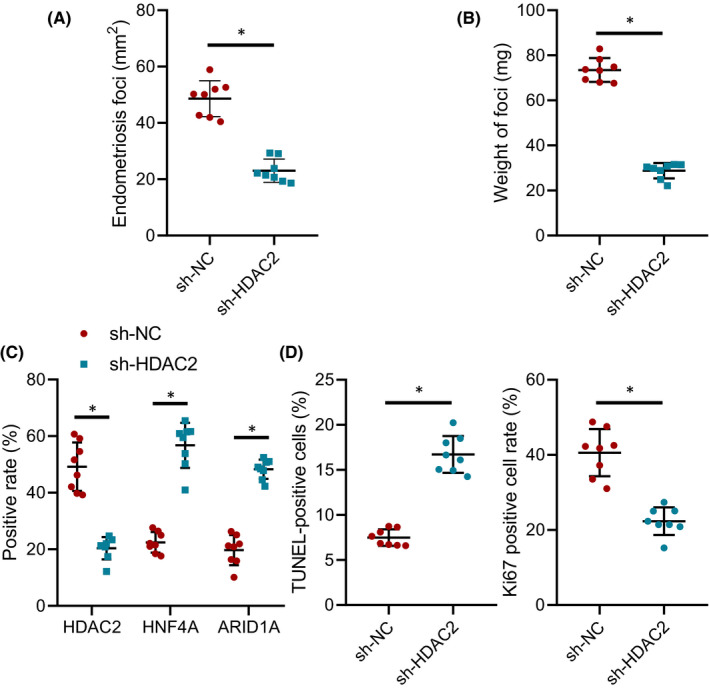
Endometriosis is repressed in vivo by silencing HDAC2. (A) The area of endometriosis tissues of mice in each group. (B) Endometriosis tissue weight of mice in each group. (C) The expression of HDAC2, HNF4A and ARID1A in endometriosis tissues of each group analysed by immunohistochemistry. (D) Ki‐67 expression measured by immunohistochemistry and apoptosis detected by TUNEL staining in each group. Data were shown as median ± standard error of mean (*n* = 8). *p < 0.05 vs. the sh‐NC group. *n* = 8 mice/group. The experiment was repeated three times independently

In order to explore the effect of HDAC2 on endometriosis in vivo, the mouse model of endometriosis was established. As shown in Figure S1, a representative mouse showed 4 stitched fragments of tissues, resulting in 3 lesion sites filled with liquid on the peritoneum wall. Then, HDAC2 was silenced. The results showed that compared with the sh‐NC group, endometriosis mice in the sh‐HDAC2 group had distinct reduction of the area and weight of endometriosis tissues (Figure 6A,B). Based on immunohistochemistry results, in contrast to the sh‐NC group, HNF4A and ARID1A protein expression in the sh‐HDAC2 group was substantially augmented whereas HDAC2 expression was diminished (Figure 6C). Immunohistochemistry and TUNEL staining manifested that compared with the sh‐NC group, Ki‐67 protein expression in the sh‐HDAC2 group was conspicuously declined in endometriosis tissues, and the apoptosis rate was evidently elevated (Figure 6D). To sum up, silencing HDAC2 inhibited endometriosis by influencing the HNF4A/ARID1A axis in vivo.

## 4 DISCUSSION

As a frequent and chronic illness in young women, patients with endometriosis have painful symptoms, and the advanced endometriosis may result in gynaecological malignancies like ovarian cancer and other complications, including infertility.^13^ Furthermore, the gold standard for endometriosis treatment is surgery, but surgery contributes to a great morbidity and cost burden for patients.^14^ Based on this, there is an ongoing need for understanding molecular mechanism underlying endometriosis to provide a novel target for endometriosis treatment. Thus, our study was designed to explore the role of HDAC2 in endometriosis and the related specific mechanism. Consequently, our data unravelled that silencing HDAC2 increased HNF4A expression through inhibition of deacetylation to upregulate ARID1A, thus preventing endometriosis.

The microarray‐based analysis in our study indicated that upregulated HDAC2 was a key gene for endometriosis, and tissue experiments confirmed the upregulation of HDAC2 in endometriosis tissues. In consistent with this finding, existing research has displayed that HDAC2 expression was higher in endometriotic cells than in endometrial stromal cells.^7^ Notably, the data in our research also exhibited that HDAC2 silencing repressed cell proliferation and invasion but accelerated cell apoptosis in endometriosis cell line hEM15A. In line with our results, Li et al. found that HDAC2 silencing evidently depressed cell proliferation and facilitated cell apoptosis in oesophageal squamous cell carcinoma EC9706 cells.^15^ In addition, another research unveiled that HDAC2‐targeting shRNA caused suppression of the in vitro migration and invasion ability of colorectal cancer cells, which was concordant with our finding.^16^ Also, ectopic expression of HDAC2 could lead to facilitation of the proliferation, migration and invasion of human trophoblast cells HTR‐8/SVNEO.^17^

Importantly, it was depicted in a prior research that HDAC2 overexpression augmented the deacetylation of HNF4A during Alzheimer's disease,^8^ which partially supported our results that HDAC2 repressed HNF4A expression through deacetylation during endometriosis. Moreover, our data further ascertained that HNF4A was downregulated in endometriosis tissues and that HNF4A silencing enhanced hEM15A cell proliferation and invasion but blocked cell apoptosis. Corroborating finding are identified in a previous study, which demonstrated that HNF4A is involved in the pathogenesis of endometriosis.^9^ Moreover, it was reported in a prior research that HNF4A deficiency diminished renal cell carcinoma cell proliferation, migration and invasion.^18^ Moreover, HNF4A upregulation could trigger inhibition of hepatocellular carcinoma proliferation.^19^ Also, it has been clarified that enhancement of hepatocyte proliferation correlated to downregulated HNF4A.^20^ The research conducted by Yao et al. detected the accelerated apoptosis of colon carcinoma cells after overexpressing HNF4A.^21^ These researches partially supported our results of enhanced hEM15A cell proliferation and invasion and repressed cell apoptosis after silencing HNF4A.

It has been widely recognized that HNF4A was a transcription factor to induce the expression of downstream factors. For example, a prior study showed that HNF4A facilitated transcriptional activation of many downstream targets, like HNF1A and factors of interleukin signalling pathway in gastrointestinal adenocarcinoma cells.^10^ Interestingly, our bioinformatics analysis uncovered that HNF4A enriched in ARID1A promoter region to activate ARID1A expression. Further analysis in our study exhibited the downregulation of ARID1A in endometriosis tissues, which was in coincidence with the finding in the research conducted by Xie et al.^12^ Furthermore, cell experiments in this research displayed elevation of proliferation and invasion but decrease of apoptosis of hEM15A cells following ARID1A silencing. In line with our finding, a prior study discovered that ARID1A overexpression diminished ovarian granulosa cell proliferation but augmented cell apoptosis in polycystic ovary syndrome.^22^ Another existing study also revealed that silencing of ARID1A by small interfering RNA induced proliferation, migration and invasion in CNE1 and HNE1 cells.^23^

## 5 CONCLUSION

**FIGURE 7:**
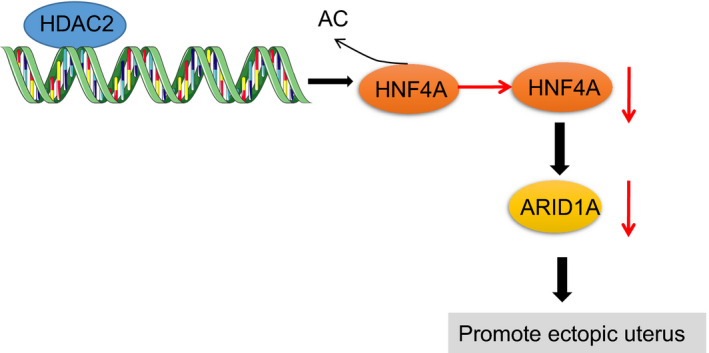
Molecular mechanism of the HDAC2/HNF4A/ARID1A axis involved in endometriosis. HDAC2 inhibited HNF4A through deacetylation, thus diminishing ARID1A expression to promote endometriosis

Collectively, the current findings elicited that the silencing of HDAC2 might reduce the proliferation and invasion but enhance apoptosis of endometriosis cells to prevent endometriosis by activating the HNF4A/ARID1A axis (Figure 7), which might present a promising therapeutic strategy for the treatment of endometriosis. However, considering the limitations of our study, more extensive research is required to investigate the specific mechanism of HDAC2, with eventual translation to clinical trials.

## CONFLICTS OF INTEREST

The authors declare no conflicts of interest.

## AUTHOR CONTRIBUTION

**Hong Mai:** Conceptualization (lead); Data curation (equal); Formal analysis (equal); Investigation (lead); Methodology (lead); Writing‐original draft (lead); Writing‐review & editing (equal). **Yan Liao:** Conceptualization (supporting); Data curation (equal); Formal analysis (equal); Investigation (supporting); Methodology (supporting); Project administration (lead); Writing‐original draft (supporting); Writing‐review & editing (equal). **Sufang Luo:** Data curation (equal); Formal analysis (equal); Resources (equal); Software (equal); Supervision (equal); Validation (equal); Visualization (equal); Writing‐review & editing (equal). **Kaiyi Wei:** Data curation (equal); Formal analysis (equal); Resources (equal); Software (equal); Supervision (equal); Validation (equal); Visualization (equal); Writing‐review & editing (equal). **Feng Yang:** Data curation (equal); Formal analysis (equal); Supervision (equal); Validation (equal); Visualization (equal); Writing‐review & editing (equal). **Haijuan Shi:** Data curation (equal); Formal analysis (equal); Supervision (equal); Validation (equal); Visualization (equal); Writing‐review & editing (equal).

## Supporting information

Fig S1

Table S1‐S2

## Data Availability

The datasets generated and/or analysed during the current study are available from the corresponding author on reasonable request.
