# Supplementary figures and images for: Histone deacetylase HDAC2 silencing prevents endometriosis by activating the HNF4A/ARID1A axis
Source: J Cell Mol Med. 2021 Sep 29;25(21):9972–82. doi: 10.1111/jcmm.16835 (PMC8572779; doi:10.1111/jcmm.16835)

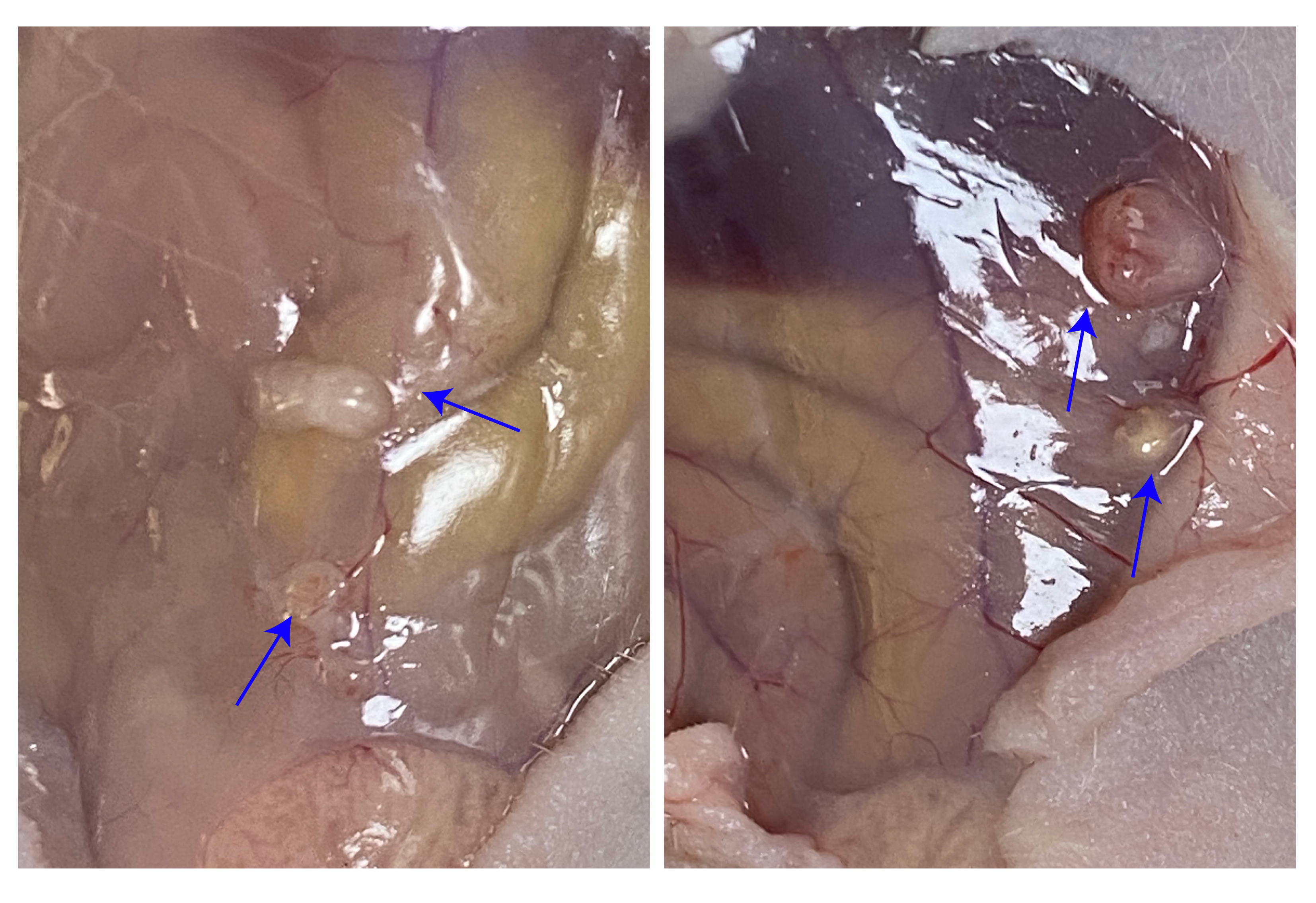

Supplement: Supplementary file 1 — Fig S1 [file JCMM-25-9972-s001.jpg]
