# Supplementary material for: Histone deacetylase HDAC2 silencing prevents endometriosis by activating the HNF4A/ARID1A axis
Source: J Cell Mol Med. 2021 Sep 29;25(21):9972–82. doi: 10.1111/jcmm.16835 (PMC8572779; doi:10.1111/jcmm.16835)
Supplement: Supplementary file 2 — Table S1‐S2 [file JCMM-25-9972-s002.docx]

**SUPPLEMENTARY TABLE 1** Primers for RT-qPCR

| Genes | Sequences (5'-3') |
| --- | --- |
| HNF4A | F: 5'-GACACAAGGCCAACGACCTA-3' |
|  | R: 5'-TCGCTTGGGTCCTCTATCCA-3' |
| ARID1 | F: 5'-TCTCTGTGGCCTYAAGCTGGA -3' |
|  | R: 5'-CCGTGTACATCTTGCCATCATAGA -3' |
| GAPDH | F: 5'-GCACCGTCAAGGCTGAGAAC-3' |
|  | R: 5'-TGGTGAAGACGCCAGTGGA-3' |

Note: RT-qPCR, reverse transcription quantitative polymerase chain reaction; F, forward; R, reverse; HNF4A, hepatocyte nuclear factor 4α; ARID1, AT-rich interactive domain 1; GAPDH, glyceraldehyde-3-phosphate dehydrogenase.

**SUPPLEMENTARY TABLE 2** Binding sites of HNF4A to ARID1A promoter predicted by hTFtarget

| TF | Pattern name | Source | Sequence name | Start | Stop | Strand | Score | P value | Q value | Matched motif |
| --- | --- | --- | --- | --- | --- | --- | --- | --- | --- | --- |
| HNF4A |  | database | ARID1A_Promoter | 1404 | 1422 | + | 14.1556 | 5.53E-06 | 0.0208 | CCGCGGGTAAAGTTCACGC |
| HNF4A |  | database | ARID1A_Promoter | 1407 | 1420 | + | 13.7561 | 8.86E-06 | 0.0333 | CGGGTAAAGTTCAC |
| HNF4A |  | database | ARID1A_Promoter | 1407 | 1419 | + | 12.25 | 2.21E-05 | 0.0825 | CGGGTAAAGTTCA |
| HNF4A |  | database | ARID1A_Promoter | 1407 | 1419 | + | 11.2661 | 5.37E-05 | 0.204 | CGGGTAAAGTTCA |
| HNF4A |  | database | ARID1A_Promoter | 1070 | 1084 | + | 11.0204 | 5.56E-05 | 0.11 | CTGCCCTCTGACTTT |
| HNF4A |  | database | ARID1A_Promoter | 654 | 668 | + | 10.3265 | 8.05E-05 | 0.11 | TTGGACTGTGCCTTT |
| HNF4A |  | database | ARID1A_Promoter | 1406 | 1420 | - | 10.1633 | 8.76E-05 | 0.11 | GTGAACTTTACCCGC |
| HNF4A | m-dataset-1660-2 | hTFtarget | ARID1A_Promoter | 1068 | 1079 | + | 17.4079 | 3.79E-08 | 0.00015 | CTCTGCCCTCTG |
| HNF4A | m-dataset-1661-2 | hTFtarget | ARID1A_Promoter | 1068 | 1079 | + | 16.7286 | 1.07E-07 | 0.000401 | CTCTGCCCTCTG |
| HNF4A | m-dataset-1659-2 | hTFtarget | ARID1A_Promoter | 1071 | 1079 | - | 13.9857 | 2.72E-06 | 0.0103 | CAGAGGGCA |
| HNF4A | m-dataset-1663-2 | hTFtarget | ARID1A_Promoter | 1067 | 1078 | - | 14.6053 | 3.42E-06 | 0.0131 | AGAGGGCAGAGG |
| HNF4A | m-dataset-1661-2 | hTFtarget | ARID1A_Promoter | 337 | 348 | + | 14.1286 | 5.01E-06 | 0.0094 | CACTGGCCTGGG |
| HNF4A | m-dataset-1659-1 | hTFtarget | ARID1A_Promoter | 1068 | 1083 | - | 12.7823 | 1.82E-05 | 0.0685 | AAGTCAGAGGGCAGAG |
| HNF4A | m-dataset-1660-1 | hTFtarget | ARID1A_Promoter | 1408 | 1419 | + | 12.8182 | 1.95E-05 | 0.0773 | GGGTAAAGTTCA |
| HNF4A | m-dataset-1671-1 | hTFtarget | ARID1A_Promoter | 1406 | 1419 | - | 12.5455 | 2.12E-05 | 0.0788 | TGAACTTTACCCGC |
| HNF4A | m-dataset-1652-1 | hTFtarget | ARID1A_Promoter | 1408 | 1419 | + | 12.5714 | 2.17E-05 | 0.0849 | GGGTAAAGTTCA |
| HNF4A | m-dataset-1668-1 | hTFtarget | ARID1A_Promoter | 1406 | 1419 | + | 11.8636 | 2.99E-05 | 0.117 | GCGGGTAAAGTTCA |
| HNF4A | m-dataset-1663-1 | hTFtarget | ARID1A_Promoter | 1408 | 1419 | + | 12.1667 | 3.08E-05 | 0.117 | GGGTAAAGTTCA |
| HNF4A | m-dataset-1670-1 | hTFtarget | ARID1A_Promoter | 1406 | 1419 | - | 11.5429 | 3.94E-05 | 0.143 | TGAACTTTACCCGC |
| HNF4A | m-dataset-1649-1 | hTFtarget | ARID1A_Promoter | 1404 | 1422 | - | 11.3636 | 4.36E-05 | 0.165 | GCGTGAACTTTACCCGCGG |
| HNF4A | m-dataset-1664-1 | hTFtarget | ARID1A_Promoter | 1405 | 1419 | + | 11.1364 | 4.60E-05 | 0.178 | CGCGGGTAAAGTTCA |
| HNF4A | m-dataset-1653-1 | hTFtarget | ARID1A_Promoter | 1405 | 1419 | + | 10.8 | 4.73E-05 | 0.121 | CGCGGGTAAAGTTCA |
| HNF4A | m-dataset-1651-1 | hTFtarget | ARID1A_Promoter | 1404 | 1419 | + | 10.7879 | 5.00E-05 | 0.141 | CCGCGGGTAAAGTTCA |
| HNF4A | m-dataset-1660-2 | hTFtarget | ARID1A_Promoter | 337 | 348 | + | 11.8289 | 5.00E-05 | 0.0987 | CACTGGCCTGGG |
| HNF4A | m-dataset-1661-1 | hTFtarget | ARID1A_Promoter | 1404 | 1419 | - | 11.1463 | 5.44E-05 | 0.102 | TGAACTTTACCCGCGG |
| HNF4A | m-dataset-1661-1 | hTFtarget | ARID1A_Promoter | 655 | 670 | + | 11.1138 | 5.55E-05 | 0.102 | TGGACTGTGCCTTTTC |
| HNF4A | m-dataset-1648-1 | hTFtarget | ARID1A_Promoter | 1405 | 1420 | - | 10.8636 | 6.02E-05 | 0.157 | GTGAACTTTACCCGCG |
| HNF4A | m-dataset-1653-1 | hTFtarget | ARID1A_Promoter | 655 | 669 | - | 10.2714 | 6.12E-05 | 0.121 | AAAAGGCACAGTCCA |
| HNF4A | m-dataset-1654-1 | hTFtarget | ARID1A_Promoter | 655 | 668 | - | 10.3816 | 6.16E-05 | 0.149 | AAAGGCACAGTCCA |
| HNF4A | m-dataset-1651-1 | hTFtarget | ARID1A_Promoter | 655 | 670 | - | 10.0758 | 7.13E-05 | 0.141 | GAAAAGGCACAGTCCA |
| HNF4A | m-dataset-1672-1 | hTFtarget | ARID1A_Promoter | 655 | 667 | + | 10.6515 | 7.41E-05 | 0.281 | TGGACTGTGCCTT |
| HNF4A | m-dataset-1654-1 | hTFtarget | ARID1A_Promoter | 1406 | 1419 | + | 9.98684 | 7.49E-05 | 0.149 | GCGGGTAAAGTTCA |
| HNF4A | m-dataset-1659-2 | hTFtarget | ARID1A_Promoter | 340 | 348 | - | 11.3286 | 8.24E-05 | 0.156 | CCCAGGCCA |
| HNF4A | m-dataset-1661-1 | hTFtarget | ARID1A_Promoter | 333 | 348 | + | 10.4065 | 8.60E-05 | 0.106 | GGAACACTGGCCTGGG |
| HNF4A | m-dataset-1648-1 | hTFtarget | ARID1A_Promoter | 654 | 669 | + | 10.1818 | 8.82E-05 | 0.157 | TTGGACTGTGCCTTTT |
| HNF4A | m-dataset-1659-1 | hTFtarget | ARID1A_Promoter | 1407 | 1422 | + | 10.1613 | 9.71E-05 | 0.182 | CGGGTAAAGTTCACGC |
